# PPAR*γ* in Bacterial Infections: A Friend or Foe?

**DOI:** 10.1155/2016/7963540

**Published:** 2016-09-28

**Authors:** Aravind T. Reddy, Sowmya P. Lakshmi, Raju C. Reddy

**Affiliations:** ^1^Department of Medicine, Division of Pulmonary, Allergy and Critical Care Medicine, University of Pittsburgh School of Medicine, Pittsburgh, PA 15213, USA; ^2^Veterans Affairs Pittsburgh Healthcare System, Pittsburgh, PA 15240, USA

## Abstract

Peroxisome proliferator-activated receptor *γ* (PPAR*γ*) is now recognized as an important modulator of leukocyte inflammatory responses and function. Its immunoregulatory function has been studied in a variety of contexts, including bacterial infections of the lungs and central nervous system, sepsis, and conditions such as chronic granulomatous disease. Although it is generally believed that PPAR*γ* activation is beneficial for the host during bacterial infections via its anti-inflammatory and antibacterial properties, PPAR*γ* agonists have also been shown to dampen the host immune response and in some cases exacerbate infection by promoting leukocyte apoptosis and interfering with leukocyte migration and infiltration. In this review we discuss the role of PPAR*γ* and its activation during bacterial infections, with focus on the potential of PPAR*γ* agonists and perhaps antagonists as novel therapeutic modalities. We conclude that adjustment in the dosage and timing of PPAR*γ* agonist administration, based on the competence of host antimicrobial defenses and the extent of inflammatory response and tissue injury, is critical for achieving the essential balance between pro- and anti-inflammatory effects on the immune system.

## 1. Introduction

The family of transcription factors designated peroxisome proliferator-activated receptors (PPARs) has long been studied for its role in regulation of lipid and glucose metabolism [1, 2]. More recently, PPARs' role in immunoregulation has been recognized and is the subject of intense investigation [1, 2]. PPARs are expressed by a variety of cells of the immune system including monocytes, macrophages, B and T lymphocytes, natural killer cells, dendritic cells, neutrophils, eosinophils, and mast cells [1]. In this review, we discuss the role of PPAR*γ* specifically in bacterial infections.

PPARs belong to the nuclear hormone receptor superfamily that regulates a multitude of genes [2]. There are three PPARs encoded by separate genes: PPAR*α*, PPAR*β*/*δ*, and PPAR*γ* [3]. The three PPARs differ in their structure, function, and tissue distribution [4]. PPAR*γ* has received significant attention as a key regulator of adipocyte differentiation as well as glucose and lipid homeostasis [2, 4]. PPAR*γ* can be transcribed from three distinct mRNAs, *γ*1, *γ*2, and *γ*3, based on sites of transcription initiation and splicing [4–6]. However, there are only two protein isoforms, PPAR*γ*1 and PPAR*γ*2, as translation of *γ*1 and *γ*3 mRNAs results in indistinguishable proteins [6]. PPAR*γ*1 is the predominant isoform [5]. Whereas expression of PPAR*γ*2 and PPAR*γ*3 mRNAs is restricted [6], PPAR*γ*1 mRNA is expressed fairly ubiquitously [4].

A variety of ligands, natural and synthetic, are capable of stimulating PPAR*γ* activity. Natural PPAR*γ* ligands include saturated and unsaturated fatty acids, eicosanoid derivatives such as 15-deoxy-Δ^12,14^-prostaglandin J_2_ (15d-PGJ_2_), and nitrated fatty acids such as nitrated linoleic and oleic acids [4, 7]. Synthetic PPAR*γ* agonists are represented by thiazolidinediones (TZDs) such as pioglitazone, rosiglitazone, troglitazone, and ciglitazone. In addition, some nonsteroidal anti-inflammatory drugs such as indomethacin, fenoprofen, and ibuprofen can activate PPAR*γ*, although their binding affinity is lower than that of TZDs. In the absence of these agonists, PPAR*γ* remains inactive, bound to a series of corepressors. Upon ligand activation, these corepressors are displaced, allowing PPAR*γ* to heterodimerize with retinoid X receptors and initiate transcriptional control by binding to specific peroxisome proliferator response elements in the promoter regions of target genes [4]. PPAR*γ* agonists may have other activities, though. For example, pioglitazone has been shown to alter mitochondrial function in a PPAR*γ*-independent manner [8] and nitrated fatty acids are electrophiles that alkylate and may inactivate target proteins [9, 10]. These off-target effects may be either helpful or harmful, depending on the context. Additionally, PPAR*γ* agonists are known to upregulate the receptor's expression, which may render the effects of repeated dosing greater than would be anticipated from single-dose results [11, 12].

Recognition that PPAR*γ* is expressed by a variety of immune cells stimulated interest in its immunoregulatory function, especially its anti-inflammatory role [13]. Involvement of PPAR*γ* in several leukocyte functions supports its prominent role in immunoregulation [13, 14]. Protein and mRNA expression and activity of PPAR*γ* are altered during many inflammatory conditions, and such alterations appear to be a significant factor in the pathogenesis of some diseases [15].

We are surrounded by a variety of microbial species and are constantly interacting with them throughout our lives. Some of these microorganisms are commensal or even beneficial, while others are pathogens that can cause significant morbidity and mortality. The innate immune system, characterized by secretion of proinflammatory cytokines and antimicrobial molecules and recruitment of phagocytes, is a major mediator of resistance to infection by pathogenic bacteria. Compromised or dysregulated immunity can allow development of major illnesses requiring therapeutic intervention, yet in some cases inflammatory responses themselves can become life-threatening. Although many infectious diseases can be controlled by antibacterial drugs, antimicrobial resistance poses a significant threat to our healthcare system worldwide, compromising therapy, complicating treatment, increasing mortality, and resulting in substantial financial costs [16]. Drugs with novel mechanisms of action are therefore urgently needed. Recent advances in our understanding of PPAR*γ*'s role in immunity, infection, and inflammation, as discussed below, offer the opportunity for intervention with a novel approach to bacterial infections. Appropriate antimicrobial therapy will continue to be the standard of care, but adjunctive use of PPAR*γ* agonists or antagonists may reduce the required antibiotic dosages and improve outcomes.

Human disease is complex, however, and effects on one cell type may be beneficial while those on another may tend to exacerbate the disease. Outcomes may also depend crucially on the exact nature of the disease—not only pathogen but also the state of disease development and potential comorbidities. Nevertheless, despite these complexities and resulting uncertainties, evidence supports the desirability of further investigation of PPAR*γ* ligands as potential adjunctive therapy in many infectious diseases.

## 2. PPAR*γ*: A Friend

The positive effect of PPAR*γ* (and/or its ligands) in bacterial infections, especially its anti-inflammatory effects via inhibition of proinflammatory molecules such as IL-6, TNF-*α*, IL-1*β*, and IL-12, has been well documented. In the* ex vivo* study by Aronoff et al., troglitazone, rosiglitazone, and 15d-PGJ_2_ increased the Fc*γ* receptor-mediated phagocytosis of* Klebsiella pneumoniae* as well as that of IgG-opsonized nonphysiological targets by primary lung macrophages abundantly expressing PPAR*γ* [17]. This effect appears to be mediated through PPAR*γ*, demonstrating the role of PPAR*γ* in pathogen clearance during bacterial infections. This phagocytic role of PPAR*γ* is in line with other studies showing that PPAR*γ* activation increases expression of CD36 cell surface receptors and uptake of apoptotic neutrophils by macrophages, a process critical for resolution of inflammation [18, 19]. Likewise, Stegenga et al. reported that the PPAR*γ* ligand ciglitazone alleviates* Streptococcus pneumoniae*-induced lung inflammation in mice by suppressing bacterial outgrowth and proinflammatory cytokine secretion, thereby improving survival of the infected animals [20]. Interestingly, however, contrary to previous findings [17], Stegenga et al. observed no ciglitazone-induced increase in* in vitro* phagocytosis or killing ability of alveolar macrophages in response to* S. pneumoniae* infection [20]. This discrepancy may reflect differences in agonists and pathogens used as well as the cell types employed in their studies. Nevertheless, these studies highlight the role of PPAR*γ* activation in reducing inflammation and improving pathogen clearance.

Anti-inflammatory effects of PPAR*γ* activation are not confined to bacterial infections of the lungs. In a study using a mouse model of central nervous system infection by* Staphylococcus aureus*, which is associated with brain abscesses in humans, ciglitazone reduced the expression of proinflammatory mediators as well as iNOS and inhibited microglia/macrophage activation. The authors of the study noted that ciglitazone's ability to suppress proinflammatory mediator secretion is only partial, a significant observation as complete absence of proinflammatory responses would result in persistence of bacteria in the brain parenchyma and therefore would be detrimental to survival of the infected animals. Another key finding in this study was that ciglitazone is capable not only of preventing microglial activation when administered prophylactically but also of dampening the activity of microglia that have already been stimulated by on-going bacterial infections. This is clinically relevant and important because typically patients seeking treatment for brain abscess would already exhibit inflammatory central nervous system responses. In addition to attenuation of microglial response, ciglitazone-treated animals show reduced bacterial burdens, probably due to the enhanced microglial phagocytic ability that was observed. Moreover, ciglitazone accelerates brain abscess encapsulation, as evidenced by the increased deposition and compact organization of fibronectin as well as the early emergence of *α*-smooth muscle actin-expressing myofibroblasts associated with development of the capsule, which could prevent further dissemination of the pathogens [21]. Altogether, these observations provide evidence that PPAR*γ* activation by synthetic agonists is an attractive therapeutic intervention for brain abscesses since it is capable of achieving a balance between effective clearance of pathogen and minimal damage to the brain tissue.

PPAR*γ*'s anti-inflammatory function is also prominent in chronic granulomatous disease (CGD), an inherited disorder in which phagocytes' defective ability to kill certain infectious pathogens results in chronic and recurrent infections and inflammation. In a mouse model of CGD, macrophages demonstrate reduced PPAR*γ* expression and activation and impaired efferocytosis of apoptotic neutrophils during zymosan-induced acute inflammation [22]. Monocytes from human CGD patients similarly show defective efferocytosis [23]. Furthermore, neutrophils and monocytes/macrophages from these CGD mice as well as monocytes from human CGD patients exhibit defects in PPAR*γ*-dependent production of mitochondrial reactive oxygen species (ROS) that contribute to bacterial killing [24]. These defects can be largely restored by PPAR*γ* activation with pioglitazone, given prophylactically or during preexisting inflammation [22–24], providing further evidence for the antibacterial effect of PPAR*γ* activation. Importantly, the authors showed that pioglitazone is capable of enhancing CGD phagocytes' ability to clear pathogens such as* S. aureus* and* Burkholderia cepacia*, restoring host defense against these pathogens [24]. Furthermore, the PPAR*γ* agonist pioglitazone produced marked clinical improvement in a 5-month-old boy with CGD and multiple severe infections [25]. Significantly improved ROS production was associated with reductions in pathogen burden and improvements in overall clinical condition that allowed curative hematopoietic stem cell transplantation. Although this reflects an unusual setting, these direct clinical results support the ability of PPAR*γ* agonists to upregulate pathogen killing and clearance.

Infections by a variety of bacteria can result in sepsis, in which blood-borne toxins lead to an exaggerated and dysregulated inflammatory response that frequently results in tissue injury [26]. In severe sepsis, potentially lethal septic shock and multiple organ failure become strong possibilities [27]. PPAR*γ* signaling has shown a protective effect in multiple models of sepsis. In the mouse model of lipopolysaccharide- (LPS-) induced sepsis involving pulmonary inflammation and injury, endothelial cell PPAR*γ* (ePPAR*γ*) deficiency intensifies the tissue injury with increased pulmonary edema and capillary permeability, elevated ROS and cytokine/chemokine production, infiltration of neutrophils to the lungs, and expression of inflammation-associated adhesion molecules such as ICAM-1 and PECAM-1. This exacerbation of inflammatory responses in ePPAR*γ*-deficient mice is due to enhanced toll-like receptor-4 (TLR4) expression in the lung tissues and upregulation of TLR4 downstream signaling including the NF-*κ*B pathway [26]. TLR4 signaling has been shown to play a key role in modulating inflammation/sepsis [28, 29]. In addition to the effects of PPAR*γ* agonists reported by others, Reddy et al. observed that physiologically relevant concentrations of 10-nitro-oleic acid reduce LPS-induced transcription of many inflammatory markers and inhibit neutrophil transmigration* in vitro* [26].

The protective effect of PPAR*γ* activation is also demonstrated in mouse and rat models of polymicrobial sepsis using cecal ligation and puncture (CLP) [27, 30]. Zingarelli et al. found that rats subjected to CLP exhibit reduced PPAR*γ* expression in the lungs and thoracic aortas, increased circulating neutrophils accompanied by reduction in lymphocytes, and increased accumulation of neutrophils in multiple vital organs. Elevated levels of mediators of sepsis-associated vascular dysfunction and hypotension were also detected. These cellular and molecular changes were shown to reflect upregulation of the proinflammatory transcription factors NF-*κ*B and AP-1. 15d-PGJ_2_ and ciglitazone prolong the animals' survival, reversing the sepsis-associated proinflammatory events and improving arterial blood pressure [27]. Likewise, in mice experiencing polymicrobial sepsis, pioglitazone reduces bacterial burden at the site of infection (the peritoneum) and in the blood and alleviates edema and capillary congestion at target tissues such as the lungs by reducing neutrophil infiltration and cytokine accumulation. Survival rate of septic mice consequently improves. The authors found that PPAR*γ* activation exerts its protective effect against bacterial sepsis via an IL-10-dependent reduction in expression of MyD88, a critical downstream component of the TLR pathway [30].

Remarkably, several of these studies [20, 21, 30] report that PPAR*γ* agonists exhibit both anti-inflammatory and antibacterial properties, two seemingly contradictory effects. Mechanisms underlying this unique characteristic of PPAR*γ* agonists are currently unclear. It is possible that these two properties are the results of two distinct activities of the drugs. Alternatively, limited inflammatory response may simply reflect improved pathogen clearance and thus reduced inflammatory stimulus [31]. Further research is needed to address this question.

Regardless, in bacterial infections where optimal pathogen clearance and prevention of excessive inflammation are equally critical for the health and survival of patients, PPAR*γ* agonists offer new therapeutic strategies. This may be particularly true for lung infections due to effects on resident alveolar macrophages. PPAR*γ* activation reduces the ability of inflammation to stimulate alveolar macrophage switching from an anti-inflammatory to a proinflammatory state yet simultaneously increases macrophage phagocytosis of both opsonized and unopsonized particles [32]. Differentiation of monocytes into alveolar macrophages is associated with appearance of PPAR*γ*, and macrophage-specific PPAR*γ* knockout is associated with mild steady-state inflammation in lungs but not elsewhere [33]. Additionally, absence of macrophage PPAR*γ* led to reduced bacterial clearance and increased mortality following* S. pneumoniae* infection.

Lastly, adding a new layer to PPAR*γ*'s involvement in bacterial infections is the study by Kelly et al. Here, the authors showed that* Bacteroides thetaiotaomicron*, a commensal bacterium prevalent in the human gut microflora, blocks the dysfunctional acute inflammatory response to infection by pathogenic* Salmonella enterica* by inducing binding of PPAR*γ* to the NF-*κ*B RelA subunit and their joint nuclear export and cytosolic localization, thereby inhibiting the consequent transcription of proinflammatory IL-8. Intestinal structure is mostly preserved in rats infected with both bacteria, compared to the animals exposed to* S. enterica* alone, providing evidence for the attenuation of inflammation. Importantly, this anti-inflammatory function of* B. thetaiotaomicron* is dependent on PPAR*γ*, as RNAi-mediated reduction in PPAR*γ* expression abolishes the inhibitory effect on IL-8 [34]. Thus, not only host defense but also bacteria themselves can engage PPAR*γ* for its anti-inflammatory functions during pathogenic bacterial infections.

## 3. PPAR*γ*: A Foe

Whereas the above studies present clear evidence of the protective role of PPAR*γ* and/or its agonists in bacterial infections, other research shows that PPAR*γ* expression/activation is harmful for the host, in at least two distinct fashions.

PPAR*γ* activation triggers apoptosis in a variety of leukocytes, which can dampen the host immune response during bacterial infections. Pioglitazone has been shown to induce caspase-3- and caspase-9-dependent apoptosis in macrophage-like cells derived from a human monocyte cell line. This effect is reversed by a functionally selective PPAR*γ* antagonist, GW9662, supporting its PPAR*γ* dependence [35]. Likewise, rosiglitazone, troglitazone, and ciglitazone induce and GW9662 and another PPAR*γ* antagonist, BADGE, block apoptosis of human leukemia cells in a caspase-3-dependent manner [36]. PPAR*γ* activation with 15d-PGJ_2_, troglitazone, or ciglitazone is likewise antiproliferative and proapoptotic in multiple mouse B lymphoma cell lines representing different stages of maturation and in human B lymphocytes and B lymphoma cells [37, 38]. Tautenhahn et al. observed that ciglitazone treatment causes apoptosis in phytohemagglutinin-stimulated T lymphocytes [39].

Apoptosis is thought to contribute to the lymphopenia seen in septic patients [40]. Indeed, T cell depletion leads to immunosuppression or immunoparalysis that underlies persistent infection and/or predisposes patients with sepsis to secondary infections and increased mortality [41]. The proapoptotic role of PPAR*γ* activation established by the studies previously mentioned potentially implicates the receptor in these effects. Soller et al. demonstrated that ciglitazone-mediated PPAR*γ* activation significantly increases apoptosis of human septic T lymphocytes, which is blocked by the PPAR*γ* antagonist SR-202. Intriguingly, sera from human septic patients seem to contain molecule(s) capable of specifically activating PPAR*γ* and inducing PPAR*γ*-dependent apoptosis of human T lymphocyte cells, although the exact identity of such molecules has yet to be determined. The authors therefore concluded that PPAR*γ* activation contributes to the lymphopenia observed during sepsis [40]. Schmidt et al. presented similar findings using the mouse CLP polymicrobial sepsis model as well as LPS-induced endotoxemia. They found that PPAR*γ* expression triggers T lymphocyte apoptosis and is associated with poor survival of mice experiencing endotoxemia or peritonitis whereas mice whose T lymphocytes lack PPAR*γ* or those treated with GW9662 show significantly fewer apoptotic T lymphocytes, reduced organ damage, and improved survival [41].

PPAR*γ* can also negatively affect hosts' response to bacterial infections by other mechanisms. PPAR*γ* activation can downregulate neutrophil migration, rolling, and adhesion, key processes during their chemotactic response to invading pathogens [42, 43]. This inhibition may reflect PPAR*γ*-dependent reduction in neutrophils' ability to adhere to fibrinogen and to polymerize actin [42] and/or suppression of ICAM-1 expression [43] and contributes to the host's failure to contain infections. 15d-PGJ_2_-induced PPAR*γ* activation also exacerbates pulmonary edema and tissue injury associated with LPS-induced endotoxemia by locally elevating chemokine and IL-1*β* expression and increasing the number of mucin-producing cells [44]. Other studies, however, have found that 15d-PGJ_2_ treatment improves survival in mouse [45] and rat [46] endotoxemia. Dose, timing, and species do not provide obvious explanations for the discrepant results.

Philipson et al. showed that abrogation of PPAR*γ* expression, by T lymphocyte specific conditional knockout or GW9662, enhances inflammatory and effector responses in the early stage of enteroaggregative* Escherichia coli* infection and improves bacterial clearance by increasing infiltration of leukocytes, including T lymphocytes, dendritic cells, and macrophages [47], implying a deleterious effect of PPAR*γ* activation/expression on host defense. These results must be interpreted with caution, however, since prolonged* E. coli* infection was seen only in mice on a protein-deficient diet. Mice on a normal diet clear the infection within 14 days without regard to PPAR*γ* deficiency. Despite this caveat, however, this group of studies suggests that PPAR*γ* may adversely affect the host's ability to combat bacterial infections.

Interestingly, some bacteria seem capable of modulating PPAR*γ* to assist their pathogenesis by manipulating its function in lipid metabolism: mycobacteria, such as those associated with tuberculosis and leprosy, use the hosts' lipids for intracellular survival and replication [1].* Mycobacterium bovis* bacillus Calmette-Guérin (BCG) infection induces PPAR*γ* expression and its nuclear localization in human monocytes and enhances lipid body formation in the activated macrophages. The PPAR*γ* agonist BRL49653 (rosiglitazone) also increases and the antagonist GW9662 blocks this lipid body biogenesis, confirming its PPAR*γ* dependence. Furthermore, the enhanced ability of macrophages to kill* Mycobacterium bovis* BCG in response to GW9662 treatment and failure of a nonpathogenic bacterium to induce PPAR*γ* expression strongly indicate that* Mycobacterium bovis* BCG specifically employs PPAR*γ* signaling for its pathogenesis [48]. Findings that lipid droplets within* Mycobacterium bovis* BCG-activated macrophages and* Mycobacterium leprae*-infected Schwann cells and macrophages are major production sites of eicosanoids, a class of PPAR*γ* agonists, further reinforce PPAR*γ*'s supportive role in survival and replication of mycobacteria [49–51].

The negative effect of PPAR*γ* activation during bacterial infections is supported by a systematic review and meta-analysis of 13 long-term randomized controlled trials of TZDs that involved 17,627 participants (8,163 receiving TZDs and 9,464 receiving control drugs) [52]. The analysis revealed that long-term (1–5.5 years) use of TZDs, compared to control drugs, significantly increases the risk of participants acquiring pneumonia or lower respiratory tract infection, some of which result in hospitalization, disability, or death [52]. It is important to note, however, that the effect was small even with prolonged treatment and that diabetic individuals, the subject population, are at increased risk of infection. Nevertheless, these results suggest caution in using TZDs in patients who may be particularly susceptible to infection.

## 4. Conclusions

In contrast to the use of antimicrobial drugs that directly target the problem's source, bacteria, treating infections with immunomodulatory agents such as PPAR*γ* ligands is more complex. The innate immune responses to invading pathogens can be divided broadly into an initial hyperinflammatory stage, termed the systemic inflammatory response syndrome, and a subsequent immunosuppressive stage called the compensatory anti-inflammatory response syndrome [53]. Thus, immunomodulatory drugs must achieve a fine balance between pro- and anti-inflammatory effects on the immune system, dampening excessive systemic inflammatory responses to prevent severe tissue damage and other complications without significantly affecting the essential ability of the host immune system to clear the infection.

We have here reviewed research investigating the effects of PPAR*γ* activation or inhibition during bacterial infections. These studies clearly show that PPAR*γ* is a double-edged sword, possessing both pro- and anti-inflammatory effects and exerting beneficial as well as harmful effects upon host defenses against pathogenic bacteria. While differences in the type of pathogens, disease models, and PPAR*γ* agonists/antagonists used in the research can explain many of the variations in results reported by different research groups, timing, the point during the host immune response at which drugs are administered, likely plays a large part in determining which PPAR*γ* agonist/antagonist effect predominates. For instance, blocking the anti-inflammatory cytokine IL-10, which is associated with reduced secretion of proinflammatory mediators, at an early stage of sepsis is detrimental to the host, whereas IL-10 suppression later in sepsis is linked with longer survival of the affected animals. Similarly, established sepsis may respond to immune-stimulating strategies but not to therapeutic interventions designed to suppress proinflammatory mediators secreted early during sepsis. In addition, in human patients, the effect of a PPAR*γ* agonist or antagonist would likely differ with the immune status of each individual—the exact pathophysiologic nature of immune imbalance—as well as other factors such as age, comorbidities, and genetic background [53]. Patients suffering damage and symptoms due to exaggerated immune response would benefit from the anti-inflammatory effect of PPAR*γ* ligands, while those experiencing immunoparalysis-induced symptoms would require the immune-enhancing effect of PPAR*γ* antagonists to alleviate damage and symptoms [54]. Thus, it is imperative to aim for a carefully defined balance between immune stimulation and immunosuppression in each patient. Close assessment of the competence of host antimicrobial defenses and the extent of inflammation and tissue injury, including measurement of mediators of the immune response, and adjustment in the dosage and timing of PPAR*γ* agonist/antagonist administration would be valuable for achieving the most desired outcome [55, 56].
